# Second-Degree Atrioventricular Block Following Acute Pregabalin Intoxication: A Case Report

**DOI:** 10.7759/cureus.101010

**Published:** 2026-01-07

**Authors:** Kais Regaieg, Sabrina Chaouch, Ilef Alila, Myriam Kallel, Bruno Pagis

**Affiliations:** 1 Intensive Care Unit, Groupement Hospitalier de Territoire (GHT) Grand Paris Nord-Est, Montfermeil, FRA

**Keywords:** atrioventricular block, cardiac intensive care, drug-induced arrhythmia, drug toxicity, pregabalin

## Abstract

Pregabalin is widely prescribed for neuropathic pain and anxiety disorders and is generally considered to have a favorable cardiovascular safety profile. However, rare cardiac conduction disturbances have been reported, mainly in overdose settings. We report the case of a 29-year-old woman admitted to the intensive care unit for toxic coma secondary to acute pregabalin intoxication, complicated by a second-degree atrioventricular block with 2:1 conduction. The patient required mechanical ventilation and transient chronotropic support with isoproterenol. Cardiac investigations were unremarkable. The clinical course was rapidly favorable after supportive management and drug discontinuation. This case highlights a rare but potentially serious cardiac adverse effect of pregabalin intoxication and emphasizes the importance of electrocardiographic monitoring.

## Introduction

Pregabalin is a structural analogue of gamma-aminobutyric acid (GABA) developed in the late 1990s and initially approved as an antiepileptic drug. It was subsequently approved for the treatment of neuropathic pain, fibromyalgia, and generalized anxiety disorder and is now widely prescribed across multiple medical specialties. Because of its efficacy and relatively favorable safety profile, pregabalin use has increased substantially worldwide over the past two decades [1].

Pharmacologically, pregabalin binds to the α2-δ subunit of voltage-gated calcium channels in the central nervous system, leading to reduced calcium influx and decreased release of excitatory neurotransmitters such as glutamate, norepinephrine, and neuropeptide substance P (tachykinin). Unlike classical GABAergic agents, it does not directly interact with GABA receptors. Pregabalin exhibits rapid gastrointestinal absorption, minimal protein binding, and high oral bioavailability (>90%) and is eliminated almost exclusively by renal excretion in unchanged form. Its elimination half-life ranges from five to seven hours in individuals with normal renal function but may be prolonged in cases of overdose or renal impairment [1].

The adverse effect profile of pregabalin has been extensively studied. The most frequently reported adverse effects are neurological and include dizziness, somnolence, ataxia, visual disturbances, and cognitive impairment. These effects are generally dose-dependent and reversible. A systematic review and meta-analysis of randomized controlled trials confirmed that neurological adverse events are the most common, whereas serious systemic complications are uncommon [2].

In recent years, cases of pregabalin overdose and intoxication have been increasingly reported, partly related to misuse, abuse, and intentional self-harm [3,4]. Most intoxications primarily result in central nervous system depression, with clinical presentations ranging from mild sedation to coma. Severe cases may be complicated by respiratory depression requiring mechanical ventilation, although outcomes are generally favorable with supportive care [3].

Cardiovascular adverse effects associated with pregabalin are considered rare. Unlike many cardiotropic drugs, pregabalin is not known to directly block cardiac sodium or potassium channels, and significant dysrhythmias are not typically expected. Nevertheless, isolated case reports have described bradycardia and atrioventricular conduction disturbances in the context of pregabalin exposure, particularly in overdose situations [5]. We report herein a case of acute pregabalin intoxication, with confirmatory elevated serum pregabalin concentrations, complicated by a second-degree atrioventricular block with 2:1 conduction in a young patient without underlying structural heart disease.

## Case presentation

A 29-year-old woman was admitted to the hospital for coma. Her medical history included major depressive disorder followed in an outpatient psychiatric setting and obesity (body mass index 36.5 kg/m²). She had no known cardiovascular disease and no prior history of conduction abnormalities. Her chronic medication consisted of pregabalin at a daily dose of 900 mg.

In the context of a marital conflict occurring during the night prior to admission, the patient reported ingestion of a higher-than-usual dose of pregabalin on the morning of admission, although the exact amount and timing could not be reliably determined. Shortly after arriving at her workplace, colleagues noticed an abnormal gait followed by a sudden loss of consciousness and collapse.

Emergency medical services found the patient comatose, with a Glasgow Coma Scale score of 3. Vital signs on initial evaluation were as follows: heart rate approximately 55 beats per minute, blood pressure 120/75 mm Hg, respiratory rate 20 breaths per minute, oxygen saturation of 88% on room air, and body temperature 36.6°C. There were no signs of trauma. She was endotracheally intubated and mechanically ventilated and subsequently admitted to the ICU.

The patient was admitted directly to the ICU. At ICU admission, the patient developed significant bradycardia, with a heart rate decreasing to approximately 45 beats per minute and blood pressure at 100/60 mmHg, corresponding to a mean arterial pressure of approximately 73 mmHg. Pulmonary examination was unremarkable under mechanical ventilation. Neurological examination showed deep sedation, with miotic, symmetrical, reactive pupils and no response to painful stimuli. No seizures or meningeal signs were observed.

Laboratory investigations performed on admission were largely unremarkable (Table 1). Serum electrolytes, renal function, liver enzymes, cardiac biomarkers, inflammatory markers, and hematological parameters were within normal limits, except for mild metabolic acidosis reflected by decreased bicarbonate levels. There was no evidence of myocardial injury or systemic inflammation.

**Table 1:** Laboratory findings at ICU admission AST: aspartate aminotransferase; ALT: alanine aminotransferase

Parameter	Value	Reference Range	Interpretation
Hemoglobin (g/dL)	12.6	12.0–16.0	Normal
Leukocytes (G/L)	9.34	4.0–10.0	Normal
Platelets (G/L)	218	150–400	Normal
Sodium (mmol/L)	139	136–145	Normal
Potassium (mmol/L)	3.7	3.5–5.1	Normal
Chloride (mmol/L)	108	98–107	Slightly increased
Bicarbonate (HCO₃⁻, mmol/L)	19.5	22.0–26.0	Decreased
Creatinine (µmol/L)	50	49–90	Normal
Urea (mmol/L)	1.5	2.5–6.4	Decreased
Total proteins (g/L)	61	64–82	Slightly decreased
Creatine phosphokinase (U/L)	77	30–190	Normal
AST (U/L)	41	15–37	Slightly increased
ALT (U/L)	52	13–56	Normal
Total bilirubin (µmol/L)	11	3–17	Normal
C-reactive protein (mg/L)	<3	<5	Normal
High-sensitivity troponin (ng/L)	<10	<14	Normal
Arterial pH	7.37	7.35–7.45	Normal
Lactate (mmol/L)	1.7	0.5–1.6	Slightly increased
Ethanol (g/L)	<0.10	0	Negative
Paracetamol (mg/L)	4	10–30	Negative
Pregabalin (mg/L)	30	2–8	Increased

Urine toxicological screening was performed using standard immunoassay techniques, including sedative-hypnotics, opioids, benzodiazepines, antidepressants, and other substances of abuse, and was negative. Blood ethanol and paracetamol levels were also negative. Plasma pregabalin concentration was measured at 30 mg/L. Chest radiography was unremarkable, and transthoracic echocardiography showed normal left ventricular systolic function without structural heart disease.

**Figure 1:**
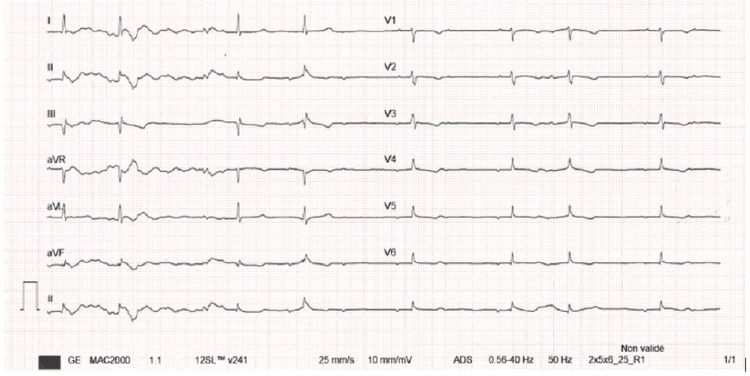
Electrocardiogram demonstrating second-degree atrioventricular block (2:1 conduction)

The initial electrocardiogram demonstrated a second-degree atrioventricular block with 2:1 conduction, associated with marked bradycardia (35-40 beats per minute) and narrow QRS complexes, without repolarization abnormalities (Figure 1). Isoproterenol was initiated as the bradycardia was considered poorly tolerated, given a trend toward decreasing blood pressure and impaired neurological status, resulting in rapid normalization of heart rate. Chronotropic support was discontinued after 12 hours, with no recurrence of conduction abnormalities.

The patient was successfully extubated on ICU day 1, with complete neurological recovery. Continuous cardiac monitoring revealed no further dysrhythmia. She was subsequently transferred to a psychiatric unit for further management.

## Discussion

The most frequently reported complications of acute pregabalin intoxication are neurological and respiratory, including somnolence, confusion, ataxia, seizures, respiratory depression, and coma, sometimes requiring mechanical ventilation [3,4]. These manifestations are largely dose-dependent and reflect the central nervous system depressant effects of pregabalin. Most cases evolve favorably with supportive care, particularly in patients with preserved renal function.

Cardiovascular complications related to pregabalin remain uncommon and are less well characterized. Pregabalin is not classically considered a cardiotoxic drug, as it does not directly block cardiac sodium or potassium channels and has no known dysrhythmic effect at therapeutic doses [1]. Nevertheless, an increasing number of case reports describe clinically significant bradyarrhythmias and atrioventricular conduction disturbances associated with pregabalin exposure, particularly in overdose settings [5].

Aksakal et al. reported a reversible complete atrioventricular block following pregabalin overdose, resolving after drug discontinuation and supportive care [5]. Schiavo et al. described first-degree atrioventricular block in a young patient receiving pregabalin, suggesting that conduction abnormalities may occur even in the absence of overdose or structural heart disease [6]. Şengüldür et al. reported PR interval prolongation following pregabalin intoxication [7]. In our patient, the plasma pregabalin concentration (30 mg/L) was markedly above the therapeutic range (2-8 mg/L) and comparable to, or exceeding, levels previously reported in severe pregabalin intoxication associated with clinically significant cardiac manifestations. Consistent with our findings, additional reports have documented significant sinus bradycardia related to pregabalin exposure, sometimes requiring temporary chronotropic support, particularly in elderly patients or those with renal impairment [8-9].

The pathophysiological mechanisms underlying these conduction abnormalities remain incompletely understood. Although pregabalin does not directly affect myocardial ion channels, its binding to the α2-δ subunit of voltage-gated calcium channels may indirectly influence atrioventricular nodal conduction. Moreover, pregabalin may modulate autonomic nervous system activity, potentially reducing sympathetic tone or enhancing parasympathetic influence, contributing to bradycardia and conduction disturbances at high plasma concentrations [1,6].

In the present case, the occurrence of a second-degree atrioventricular block with 2:1 conduction represents a rarely reported intermediate conduction abnormality. The presence of narrow QRS complexes, absence of structural heart disease, rapid response to isoproterenol, and complete reversibility after drug clearance strongly support a functional, supra-Hisian mechanism rather than intrinsic conduction system disease [10].

This case adds to the growing body of evidence suggesting that pregabalin intoxication may be associated with transient but clinically relevant cardiac conduction disturbances. It emphasizes the importance of systematic electrocardiographic monitoring in patients presenting with severe pregabalin intoxication, even in young individuals without known cardiac disease.

## Conclusions

This case highlights a rare but clinically significant cardiac complication of acute pregabalin intoxication. Although pregabalin is generally regarded as safe from a cardiovascular perspective, high plasma concentrations may induce relevant atrioventricular conduction disturbances, even in young patients without underlying heart disease. Systematic electrocardiographic assessment should therefore be performed in patients presenting with severe pregabalin intoxication. Recognizing the potentially reversible nature of these drug-induced conduction abnormalities is essential to ensure appropriate management and may help avoid unnecessary invasive interventions such as permanent pacemaker implantation.
